# Hemodynamic (fNIRS) and EEG (N200) correlates of emotional inter-species interactions modulated by visual and auditory stimulation

**DOI:** 10.1038/srep23083

**Published:** 2016-03-15

**Authors:** Michela Balconi, Maria Elide Vanutelli

**Affiliations:** 1 Research Unit in Affective and Social Neuroscience, Department of Psychology, Catholic University of Milan, Milan, Italy; 2 Department of Psychology, Catholic University of the Sacred Heart, Milan.

## Abstract

The brain activity, considered in its hemodynamic (optical imaging: functional Near-Infrared Spectroscopy, fNIRS) and electrophysiological components (event-related potentials, ERPs, N200) was monitored when subjects observed (visual stimulation, V) or observed and heard (visual + auditory stimulation, VU) situations which represented inter-species (human-animal) interactions, with an emotional positive (cooperative) or negative (uncooperative) content. In addition, the cortical lateralization effect (more left or right dorsolateral prefrontal cortex, DLPFC) was explored. Both ERP and fNIRS showed significant effects due to emotional interactions which were discussed at light of cross-modal integration effects. The significance of inter-species effect for the emotional behavior was considered. In addition, hemodynamic and EEG consonant results and their value as integrated measures were discussed at light of valence effect.

Previous research has revealed that the processing of emotional visual and auditory stimuli leads to increased activation of various cortical areas, including the amygdala, the prefrontal cortex (PFC), the dorsolateral prefrontal cortex (DLPFC) and the specific sensory areas^1^,^2^,^3^. More recent studies have identified the DLPFC as a key region in the experience and regulation of visual emotional responses^4^,^5^,^6^,^7^,^8^. Also, auditory emotional stimulation was examined, showing a similar effect to that found for the visual condition. However, in some studies which focalized on EEG frequency band analysis or event-related potentials (ERPs), it was found that frontal areas are responsive to some specific valence^9^,^10^ and, in other cases, mainly to arousal of auditory stimuli more than to their specific valence^11^.

Limited research explored the effect of combined emotional visual and auditory stimulation. A main caveat of previous studies was that they were focalized on typical human conditions or situations (stimuli with emotional value, i.e. human faces and voices)^12^,^13^,^14^,^15^, and limited research considered the effect of visual and auditory emotional stimulation in human/non-human social interactions. Some of them focalized on the empathic emotional response by humans to different species^16^,^17^, or on the brain correlates of pain perception in observing animals^18^.

Therefore, actually there is a need to improve our knowledge about the nature and the cortical correlates of the emotional behavior in response to inter-species emotional condition. In the present study we specifically considered the cortical response to emotional interactions induced by visual and visual-auditory stimulation more directly related to inter-species relationships, where an emotionally positive or negative human-animal interactions were represented. Indeed the great majority of previous studies focused only on human-human context, even if we don’t exclusively interact with other people: in fact, as part of our everyday life, we share our social contexts with also non-human animals. Few previous studies explored emotions in inter-species contexts, examining the differences between the emotional response for other humans or animals, but none of them, at our knowledge, considered the social meaning of intra- and inter-species contexts. More specifically, the contribution of specific brain areas implicated in human/non-human interactions was scarcely considered. The comparison between different species could or could not support the homogeneity of the emotional behavior and of the emotional brain response to the inter-species interactional context. In addition, the specificity of visual and auditory stimulation was not explored, since many studies focused on each stimulation condition separately, or took into consideration only the inter-species condition^19^.

Previous findings on human-human interactions provided compelling evidence of an early integration of visual (i.e. face) and auditory (i.e. voice) information while processing emotional features^20^,^21^,^22^,^23^. Both behavioral and electroencephalographic (ERPs) data revealed some, although non-identical, patterns of cross-modal influences: for example, a modulation of P200 ERP-component suggested the presence of a relatively early integration process^24^. Also, congruous and incongruous cross-modal stimulation was found to induce specific and distinct effects^22^,^23^,^25^. The largest EEG alpha-power-density was observed for the sound conditions, with intermediate values for the picture conditions, and the lowest ones for the combined conditions, thus indicating the strongest activation (alpha decreasing) in the combined condition within a distributed emotion and arousal network comprising frontal, temporal, parietal and occipital neural structures^26^. Also, strong similarities in alpha and beta bands during visual and audiovisual conditions were found, suggesting the intervention of a strong visual processing component in the perception of audiovisual stimuli^27^.

In addition, recent results mainly focused on visual stimulation indicated a significant and specific lateralization effect of PFC activation, based on positive (more directly processed by the left hemisphere) and negative (more directly processed by the right hemisphere) valence of emotions^28^,^29^. Indeed, the valence model supposes that cortical differences between the two hemispheres are attributable to positive vs. negative valence of emotions^29^,^30^,^31^. However, some other perspectives suggested a dichotomy on approach/avoidance motivation to emotions, the first being more left frontal-related and the second more right frontal-related^32^,^33^,^34^. Indeed, based on the approach-withdrawal model of emotion regulation, the emotional behavior should be associated with a balanced activity in the left and right frontal brain areas which can be explained in an asymmetry measurement^35^,^36^.

However, previous research did not verify simultaneously the specific visual and auditory lateralization effect based on valence, but more often only auditory^37^ or visual stimuli^4^,^38^ were used.

Among the different modalities available for monitoring brain activity, EEG/ERPs and Near-Infrared Spectroscopy (NIRS) are non-invasive and particularly well-suited for evaluating the PFC activity. In fact, although studies have provided functional images of activated brain areas associated with emotional tasks, they have seldom addressed the temporal course of such activation. Due to its fast temporal evolution and its representation and integration among widespread neural networks, emotion representation, together with its neurobiological correlates, should preferably be examined by means of imaging and EEG methods that offer good resolution in both temporal and spatial domains.

To verify these effects, we applied ERPs analysis to investigate the neural correlates in response to emotional contexts and the relation between these brain-based potentials and the simple (only visual) or cross-modal (visual and auditory) stimulation. One specific ERP deflection was analyzed, that is the N200 effect. This deflection was considered a specific marker of the emotional value, the relevance and the salience of the situation, as well as of the emotional involvement (arousal) induced by the affective condition. Indeed, the motivational significance of emotions affects subjects’ cortical responses also at longer latencies, since it was found that emotionally salient stimuli generate greater amplitudes of ERP response for N200^39^,^40^, for the positive deflection P300^41^,^42^, and also for late positivity (LPP) measures^43^,^44^,^45^.

In some studies that examined contextual emotional impact on behavior, the specific ERP component N200 was found to be directly related to the emotional content of stimuli or situations^13^,^46^,^47^,^48^,^49^. Specifically, it was previously related to the degree of the attentional and emotional relevance of the context^50^, and it was observed to be related to subjects’ emotional involvement in terms of arousal^4^,^51^. More specifically, N200 was found to be induced by the emotional cues (such as faces) more than neutral cues for explicit^51^ or implicit tasks^52^, and it was interpreted as a task-specific emotional index^53^,^54^, able to highlight the comprehension of the emotional significance of the stimulus. Indeed, this ERP component was found to be modulated by the judgment of affective arousal and valence of emotional stimuli^55^,^56^,^57^,^58^,^59^.

About the second measure, the temporal resolution of fNIRS is high enough for measuring event-related hemodynamic responses. In fact, one of the most relevant features of NIRS is its high temporal resolution compared to other imaging techniques^60^,^61^. This is an important feature for the present study, since the integration between EEG and hemodynamic measure requires an adequate comparable temporal resolution. Previous studies have provided functional images of activated areas in the brain which are associated to emotional tasks, but they have seldom addressed the temporal course of such activation. Therefore, to study the integration between electrophysiological and hemodynamic data, these specific measures seem indicated.

Although other neuroimaging techniques (such as fMRI) may offer a complete view of the cortical and sub-cortical areas implicated in emotional processing, their low temporal resolution prevents a deep exploration of the dynamic of the emotional experience. Therefore, neither the classical neuroimaging nor the electrophysiological measures seem to completely describe in depth the nature of the emotional correlates. For this reason the integration of the hemodynamic and EEG measures may offer a complete overview of the brain activity modulation with a more adequate spatial and temporal resolution^62^,^63^,^64^.

In addition, some specific areas more directly related to emotional processing, i.e. the PFC, are easily accessible for fNIRS measurements. Interestingly, recent studies using fNIRS investigating the neural correlates of emotion regulation processes also described an activation of the PFC^65^,^66^,^67^. Recently, fNIRS has been successfully applied to investigate the emotional modulation of the visual cortex^66^,^68^, but further investigations on the auditory domain are pending. It should be noted that some advantages are related to fNIRS studies with auditory stimulations^69^, whereas it can be difficult to explore the effects of emotional sounds in conventional functional Magnetic Resonance Imaging (fMRI) scanners. As shown by Plichta *et al.*^69^ the auditory cortex activation is modulated by emotionally arousing complex auditory stimuli. Consistent with this hypothesis, pleasant as well as unpleasant sounds led to higher auditory cortex activation, as compared to neutral sounds. This finding is in line with previous functional imaging studies on visual stimuli, where occipital regions were found to be more activated by emotionally arousing compared to neutral stimuli^66^.

However, at present no specific fNIRS study analyzed the PFC contribution in auditory emotional processing. In addition, no previous research by ERP and fNIRS considered the effect of auditory and visual condition together in the case emotional interactive situations where inter-species relationships are represented. Thus, the first goal of this study was to investigate the brain response to interpersonal inter-species contexts with different emotional valence by examining ERPs and fNIRS components elicited in individuals’ responses. Specifically, three different emotional situations were included: a first condition where subjects observed a conflictual and emotionally discomfortable situation (negative situations); a second condition where the subject observed a cooperative and emotionally comfortable situation (positive situations); and a third condition (control condition) where a more neutral interaction was represented (no positive or negative emotional situation). Moreover, the effect of simple visual (V) stimulation and combined visual and auditory (VU) stimulation was considered during these differently valenced situations.

We hypothesized that N200 and fNIRS-measured oxygenated haemoglobin (O2Hb) changes may be related to the emotional content of the stimuli when subjects have to observe emotional inter-species interactions. Specifically, based on previous evidence, we supposed an increased left or right PFC activity as a function of valence. Indeed we expected significant differences in response to different valenced situations: based on valence and approach/withdrawal models of emotions, a significant and consistent higher left prefrontal activation (increased O2Hb; higher N200) was supposed for positive emotional conditions, whereas a consistent higher right prefrontal activation was expected in response to negative conditions^28^,^30^. Moreover, this relationship should be accompanied by a specific stimulation effect: compared to V, VU could show an increased brain response for both ERP and fNIRS in concomitance with valence, since the cross-modal perception could increase and strength the basic effects expected for the simple emotional visual condition. Finally, a significant correlation was supposed between ERP and fNIRS components, considered as markers of electrophysiological and hemodynamic measures, respectively.

## Results

The following set of analyses was performed on the data: a first repeated measures ANOVA was applied to the ERP peak amplitude (N200); a second analysis was applied to O2Hb d values. Finally correlational analysis was applied to N200 and d measures.

A preliminary analysis tested the significant differences between the baseline (neutral) and emotional condition. For both EEG and NIRS measures the negative and positive conditions revealed significant differences compared (P ≤ 0.01) with neutral condition. Due to the systematic effect and the preliminary value of this comparison, we did not included the neutral condition in the successive analyses.

### EEG

ERPs data were entered into four-ways repeated measure ANOVAs, with factors condition (2, V-VU) × lateralization (2, left-right) × valence (2, positive-negative) × localization (3, frontal-temporo/central-parietal) applied to the peak amplitude. Type I errors associated with inhomogeneity of variance were controlled by decreasing the degrees of freedom using the Greenhouse-Geiser epsilon. Bonferroni correction was applied to the statistical data for multiple comparisons. Post hoc comparisons were successively applied to the data (contrast analyses for repeated measure ANOVA).

**Figure 1:**
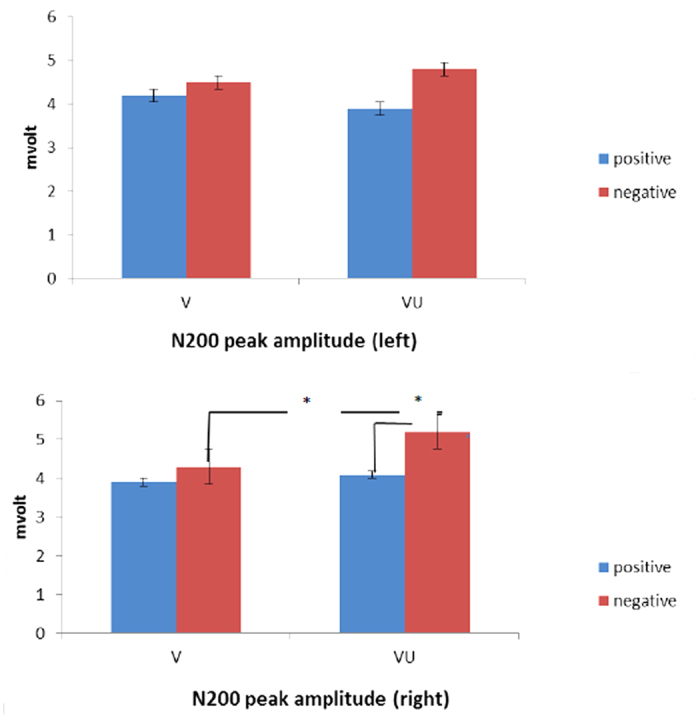
N200 peak amplitude (mvolt) for left (up) and right (down) side in response to positive and negative interactions as a function of V and VU.

As shown by ANOVA, the peak amplitude was modulated by localization (*F*(1,14) = 7.09, *P* ≤ 0.001, η^2^ = 0.34), valence (*F*(1,14) = 6.56, *P* ≤ 0.001, η^2^ = 0.33), and valence × condition × lateralization (*F*(1,28) = 9.13, *P* ≤ 0.001, η^2^ = 0.42). No other main or interaction effect was statistically significant. As observed, peak amplitude was higher for negative than positive stimuli. In addition, post-hoc comparison revealed that the frontal areas showed higher peak amplitude compared to other cortical sites: respectively compared to temporo-central (*F*(1,14) = 8.71, *P* ≤ 0.001, η^2^ = 0.39) and to parietal (*F*(1,14) = 7.56, *P* ≤ 0.001, η^2^ = 0.37) sites (Fig. 1).

In addition, about the simple effects for the three-way interaction, significant differences were observed between VU (higher peak deflection) and V in response to negative stimuli within the right side (*F*(1,14) = 6.98, *P* ≤ 0.001, η^2^ = 0.35). Secondly, in VU significant differences were found between positive and negative stimuli within the right side (*F*(1,14) = 8.87, *P* ≤ 0.001, η^2^ = 0.41), with increased peak amplitude for negative than positive stimuli.

### fNIRS

The statistical analysis was applied to d dependent measure for O2Hb concentration. Since at the analysis HHb was not significant, we report only results for O2Hb-values. D was subjected to three factor (condition, 2 × lateralization, 2 × valence, 2) repeated measures ANOVA. Data were averaged over left (Ch 1: AF3-F3; Ch2: AF3-AFF1; Ch3: F5-F3) and right (Ch4: AF4-F4; Ch5: AF4-AFF2; Ch6: F6-F4) channels.

**Figure 2:**
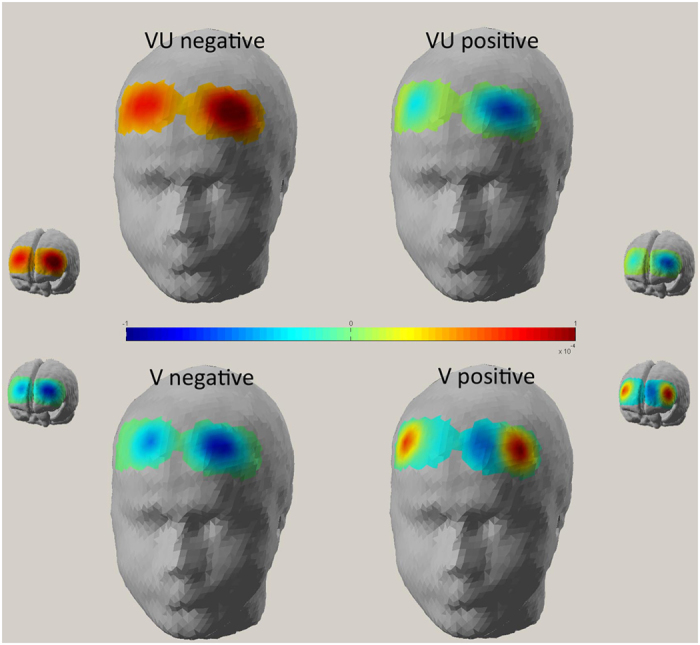
Cortical maps of O2Hb as a function of VU (up) and V (down) in response to positive (right heads) and negative (left heads) interactions.

As shown by ANOVA, interaction effect condition × valence (*F*(1,14) = 6.92, *P* ≤ 0.001, η^2^ = 0.33) was significant (Fig. 2). No other main or interaction effect was significant. Indeed, as shown by paired comparisons, for VU negative stimuli in comparison to positive stimuli revealed increased d values (*F*(1,14) = 6.79, *P* ≤ 0.001, η^2^ = 0.33). In contrast, for V positive stimuli in comparison with negative stimuli showed increased d values (*F*(1,14) = 6.13, *P* ≤ 0.001, η^2^ = 0.32).

### Correlational analysis between fNIRS and EEG

Pearson’s correlation analysis (across-subject correlations) was applied to N200 and d values. Correlational values were calculated distinctly for each condition (V/VU) emotional valence (positive/negative stimuli) and lateralization (left/right).

**Figure 3:**
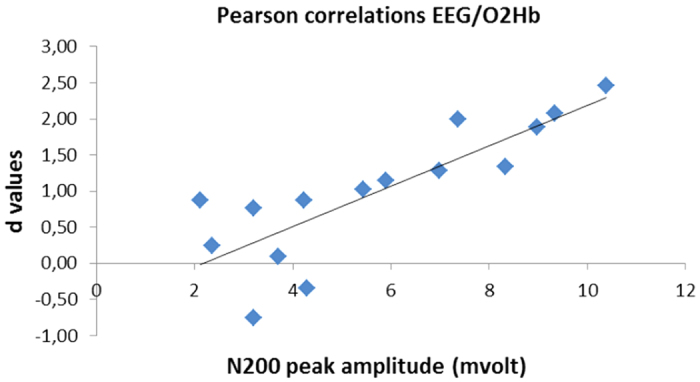
Scatterplot of EEG (N200 amplitude) and fNIRS (d values) measures in relationship with negative interactions for VU within the right side.

There was a significant positive correlation between d values and N200 for negative patterns within the right site for VU (*r* = 0.513; *P* ≤ 0.01). That is, in case of increased peak amplitude a concomitant O2Hb increasing was observed within the right hemisphere in response to negative stimuli. No other correlation was significant at the analysis (Fig. 3).

## Discussion

The present research explored the role of the prefrontal cortex while processing inter-species emotional interactions. Visual and visuo-auditory emotional stimulation was provided in different relational contexts (positive, negative and neutral). DLPFC was mainly implicated in affective response to inter-species emotional relationship. However, significant differences were found in inter-species relationships based on stimulation type, with increased cortical response for cross-modal stimulation than simple visual stimulation. Moreover, stimulus valence affected both the N200 ERP and the hemodynamic response, with a more relevant impact of the negative valence. This cortical activity was shown to be lateralized within the right hemisphere in response to negative situations. Finally, the systematic relationship between hemodynamic and EEG measures was elucidated.

In general, the present results confirmed the crucial role of the PFC in processing emotional interactions also in case of inter-species condition. Indeed, for the first time this study demonstrated the contribution of this cortical areas in response to human-non human interactions, considering both O2Hb and N200 effect. The specific DLPFC contribution in processing emotional interactions was supported by the hemodynamic (increased O2Hb) and ERP (higher N200 peak amplitude) profiles. This result confirmed previous research within the visual domain in the case of human contexts, which found that the PFC plays a crucial role in the integration of different aspects of emotional regulation by managing the cognitive control over emotional stimuli and behavior^70^,^71^,^72^,^73^,^74^. Therefore we may suggest that a specific prefrontal cortical area may mediate the emotional processing and behavior of subjects who are observing inter-subjective interactions, independently from the human specificity of such interaction. In fact, whereas in previous research only human conditions (single subjects or interactive situations) were monitored, in the present study the aspecific human-animal interactions were analyzed. In addition, previous study which included inter-species condition did not considered visuo-auditory stimulation. Therefore, a direct comparison between these two stimulation types was not considered^19^. Based on our results we may suppose that the DLPFC is involved in processing situations where emotions are represented independently from the exclusive presence of human actors^75^.

Specifically, about EEG, within this prefrontal network, the N200 amplitude appeared to be significantly modulated over the anterior brain sectors and to be valence-related. Indeed, the N200 higher amplitude within the anterior brain region was directly associated with negatively valenced situations. Based on previous results, and in accordance with its frontal localization, we may suggest that the N200 is involved in the detection and evaluation of relevant and threatening patterns^76^,^77^,^78^. This observation is in accordance with other findings which showed that the N200 is most pronounced over the frontal cortex for negative related stimuli^58^,^76^. About the fNIRS, the PFC contribution in processing emotional interactions was confirmed by the hemodynamic measure. Indeed a similar profile was observed for O2Hb, with more intense DLPFC responses for negative categories. As shown in previous research on visual stimulation, negative emotional conditions produced increased cortical response^65^,^66^,^79^,^80^,^81^, processed as being more salient for the subjective safeguard^82^. These results appear partially in contrast with a previous study, which more directly compared intra- and inter-species stimuli^19^. Indeed it was found that intra-species compared to inter-species conditions elicited higher responses for negative stimuli, whereas the opposite result was found for positive stimuli (with higher response in the case of inter-species interactions). However, also due to the specific empathic task, in that case it was supposed that in negative situations human-human interactions may produce higher controlled cortical activity than human-animal ones, because they could raise more cognitive and mediated processes to represent higher social and culture-based interactions. In addition, in the present study we did not directly compare intra- and inter-species interactions and, therefore, the direction of potential differences between these two specie-specific and specie-aspecific situations cannot be appropriately discussed. Finally, only visual stimulation was included in this previous study, choice that may prevent to produce a complete comparison between the results.

A second main factor able to affect the prefrontal response was related to stimulation type in integration with the stimulus valence, that is the presence of a simple visual emotional display or a cross-modal stimulation. Indeed, the integrated visual-auditory condition was able to induce a sort of “reinforce effect” for specific situations, that is the negative ones. Whereas the negative stimuli generally supported an increased prefrontal response, in the case of VU subjects showed a higher N200 peak amplitude and an O2Hb increasing during interactions processing compared to simple visual stimulation.

The correlational results reinforced this hypothesis. Indeed we found a higher and coherent response across EEG and fNIRS in concomitance with the cross-modal stimulation when negative situations were represented. In contrast positive valence revealed a significant effect only for the hemodynamic profile, with an O2Hb increasing for the positive category in the case of simple V. For the first time, by using two specific cortical measures, which were able to allow an adequate spatial and temporal resolution, we demonstrated the direct implication of the DLPFC in response to inter-species interactions when different stimulus modalities were considered (V and VU).

Therefore, as shown in previous studies^9^ we may suppose the auditory component may induce a higher emotional value in the subjective perception of the interactions, especially when the represented emotional content is negative and aversive. However for the first time this effect was shown in the case of cross-modal integration and not exclusively related to visual or auditory stimulation per se. Specifically, we may suppose that the negative and aggressive valence of the integrated VU conditions made the emotional relevance of those contexts as more salient. This result is consistent with the position that it may exist an approach motivational tendency toward stimuli with negative emotional valence^34^,^83^, and, more generally, an active response may be associated with approach motivation, in case of more prominent emotional cues, such as negative and unpleasant situations which the subject is processing.

This may be also due to the specificity of the inter-species context which produces the potential uncontrolled situation which the subject is faced with: in that case the unpredictable outcomes of the aggressive inter-species interactions may be evaluated as more dangerous than the positive interactions. In addition, in relation to the prefrontal localization of the cortical network, the general electrophysiological response and the hemodynamic increased responsiveness for VU in concomitance with negative patters may suggest the existence of frontally distributed vigilance mechanism activated during the detection and evaluation of potentially more dangerous emotional interactions, which is likely to be located at the extended anterior sites. That is, an attentional network involving the frontal site is argued to maintain a state of alertness when salient and more negative interpersonal conditions are encountered^84^.

It has to be noted that, compared with some previous research on neuroimaging and NIRS^66^,^85^, we found that valence was more relevant to process emotional cue, and it may be in relation to hemispheric lateralization. Indeed, about the contribution of the two hemispheres, the specific right lateralization found in response to negative stimuli, as shown by N200 peak distribution, may indicate that a clearer cortical lateralization regards largely this specific negative valence category. Indeed, this effect was observed not indistinctly, but it was noted mainly in response to certain emotional categories, such aggressive negative conditions, that is emotions with a potentially involving arousing power by the subjects^23^,^50^. To summarize, a general right/negative association was observed in the subjects and it was mainly supported by EEG modulation. That is, negative, aversive interactions showed a more consistent lateralized brain activation in comparison with other emotional categories (i.e. positive emotions).

This fact may be explained taking into account the impact of threatening and negative interactions which may be more “salient” for the subject’s safeguard, with a more specific contribution of the right DLPFC. Therefore, based on these effects, it should be noted that the valence model with an expected lateralized response (right-negative; left-positive distinction) is only partially supported by the present results. This “lateralized mechanism” may be represented as finalized to alert the emotional behavior in response to highly significant emotional situations subjects are faced with. However, due to the partial verification of the underlying valence model of emotions, future research should better explore the significance of the positive/negative valence distinction in concomitance with the approach/avoidance attitude model of emotions, to better clarify the contribution of the left vs. right hemisphere in response to emotional visual/auditory cues. In addition, the specific role that arousal may have in affecting the lateralized emotional response should be considered to explain these data more deeply^11^.

A relevant result was also the presence of a general direct link between the different levels of analysis (ERP and fNIRS measures), taking into account that EEG activity was systematically associated with the cortical hemodynamic responsiveness to the emotional situations. Indeed, important effects were derived from to the correlational analyses between hemodynamic and cortical EEG. The joined EEG-NIRS couple revealed significant linear associations between the hemodynamic O2Hb values and N200 peak amplitude. The significant and consistent positive relation between NIRS and EEG measures mainly in response to negative stimuli may suggest, from one hand a strength relation between the PFC and emotional stimuli processing, with significant effect of valence (more for negative) factor; from the other hand, it may support the consistence of these two brain measures. More generally, the simultaneous application of EEG and fNIRS was found to be particularly useful for emotional studies. Specifically, the use of fNIRS in a topographic approach for measuring responses to emotions allows to investigate regional cortical activation changes that are related to emotional manipulations in general, and to link certain EEG effects to the regional hemodynamic changes^65^,^66^. The latter strategy enabled us to investigate whether and how specific emotion-related electrophysiological effects are associated with distinct cortical activation patterns within the emotion perception network^86^.

However, some limitations of the present research should be underlined. Firstly, the limited number of subjects implicated in the study required further research to generalize the present results. Secondly, a more direct comparison between different types of relationships (i.e. intra-species and inter-species) should be included to better distinguish the two domains and to extend our results to different human-human/human-animal contexts. Finally, a complete research design, which may add to V and UV condition also a simple U condition, could furnish important elements to discriminate between simple sensory or cross-modal perception.

## Method

### Subjects

15 subjects, 8 females and 7 males (M age = 26.33; SD = 2.5; range = 23–33) participated at the experiment. All subjects were right-handed, with normal or corrected-to-normal visual acuity. Exclusion criteria were neurological or psychiatric pathologies. They gave informed written consent for participating in the study, and the research protocol was approved by the Ethical Committee institution where the work was carried out (Department of Catholic University of Milan, Italy). The experiment was conducted in accordance with the Declaration of Helsinki and all the procedure were carried out with adequate understanding of the subjects. Research Consent Form was submitted before participating in this research). No payment was provided for their performance.

### Stimuli

**Figure 4:**
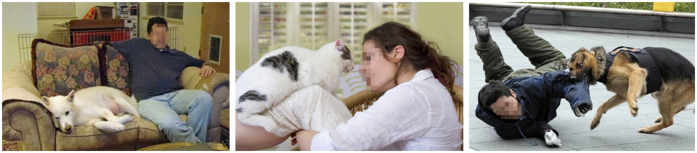
Examples of stimuli (neutral, positive, negative) (photographed by Maria Elide Vanutelli).

Subjects were required to view affective pictures depicting human-animal (for animals: cats and dogs) interactions. 48 colored pictures were selected representing positive (24) and negative (24) interactions. Positive pictures represented emotionally comfortable interactions between humans and animals; negative pictures represented emotionally uncomfortable interactions between humans animals. 24 Neutral stimuli (interactions without a specific emotional significance) were used as control condition. Other 48 pictures (positive; negative) associated with sounds which simulated animals’ positive (for cat meow/purr; for dog barks of joy) or negative (for cat growl; for dog snarl) noise (Fig. 4). Neutral condition included both visual and auditory neutral stimuli. Therefore each subject was submitted to visual (72) or visual/auditory (72) condition for a total of 144 stimuli.

All pictures had same size (14 cm in weight × 10 cm in height) and were similar for perceptual features (luminance; complexity, i.e. number of details in the scene; characters’ gender: half males and half females actors; animals’ species: half dogs and half cats). Sounds were taken from some internet databases and downloaded as wav files. They were reproduced taking into account some acoustic parameters (pitch; intensity; range) to guarantee similar profile across the noises.

A pre-experimental procedure was adopted to validate the picture/sound dataset. Each stimulus (visual or auditory) was evaluated by six judges on valence and arousal dimensions, using the Self-Assessment Manikin Scale with a five-point Likert Scale^55^,^87^. Ratings were averaged across all presented pictures/sounds for each valence.

As shown by statistical analysis (repeated measures ANOVA), both visual and auditory stimuli differed in term of valence (for all significant contrast comparisons P ≤ = 0.01), it being more positive for positive pictures/sounds than the other two categories; more negative for negative pictures/sounds than the other two categories; with intermediate values for neutral pictures/sounds than the other two categories. About arousal, positive and negative pictures/sounds were more arousing than neutral pictures/sounds. However, negative and positive stimuli did not differ in terms of arousal. The cross-modal stimulation was perceived as coherent in term of valence (negative valenced for negative stimuli combination; positive valence for positive stimuli combination).

### Procedure

**Figure 5:**
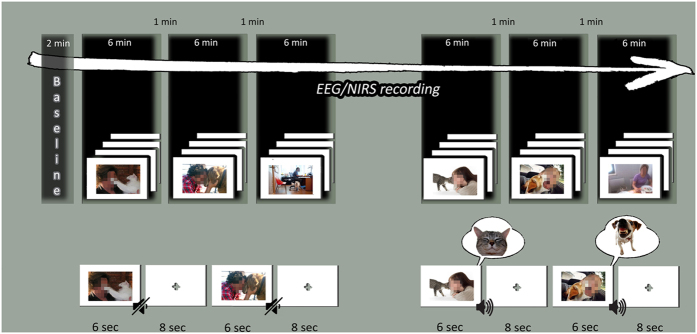
Experimental procedure (EEG and fNIRS acquisition) (photographed by Maria Elide Vanutelli).

Subjects were seated in a dimly lit room, facing a computer monitor that was placed 70 cm from the subject. Stimuli were presented using E-Prime 2.0 software (Psychology Software Tools, Inc., Sharpsburg, PA, USA) running on a personal computer with a 15-inch screen. Participants were required to process each stimulus during fNIRS/EEG measures recording, and they should attend to the pictures/sounds the entire time of exposition, focalizing on the emotional conditions which characterize the represented human actors. Subjects were submitted to V and VU blocks with a random order to avoid condition order effects (randomization of the six blocks across-subjects). Within each block, pictures or pictures/sound were displayed in a random order across-subject in the center of a computer monitor for 6 seconds, with an inter-stimulus interval of 8 seconds (Fig. 5). Auditory stimuli were reproduced by the PC loudspeakers at a listening level of approximatively 70dB. V and VU conditions were randomized across-subjects.

120 seconds resting period was registered at the beginning of the experiment before the picture/sounds series. After the experimental phase, subjects were required to rate pictures/sounds on SAM evaluating valence and arousal on a five-point Likert scale. As shown by statistical analysis (repeated measures ANOVAs) pictures/sounds differed in term of valence (for all paired comparisons P ≤ = 0.01; for visual, positive M = 4.22 SD = 0.06; negative M = 2.08 SD = 0.07; neutral M = 3.17 SD = 0.05, for auditory, positive M = 4.32 SD = 0.04; negative M = 2.13 SD = 0.05; neutral M = 3.12 SD = 0.08) and arousal (significant differences between neutral/positive and neutral/negative comparisons for both visual and auditory, P ≤ = 0.01; for visual, positive M = 4.21 SD = 0.07; negative M = 4.72 SD = 0.03; neutral M = 3.11 SD = 0.04, for auditory, positive M = 4.02 SD = 0.04; negative M = 4.80 SD = 0.08; neutral M = 3.44 SD = 0.09). In contrast no differences were found between positive and negative stimuli for both visual and auditory condition in term of arousal.

### EEG recordings and data reduction

**Figure 6:**
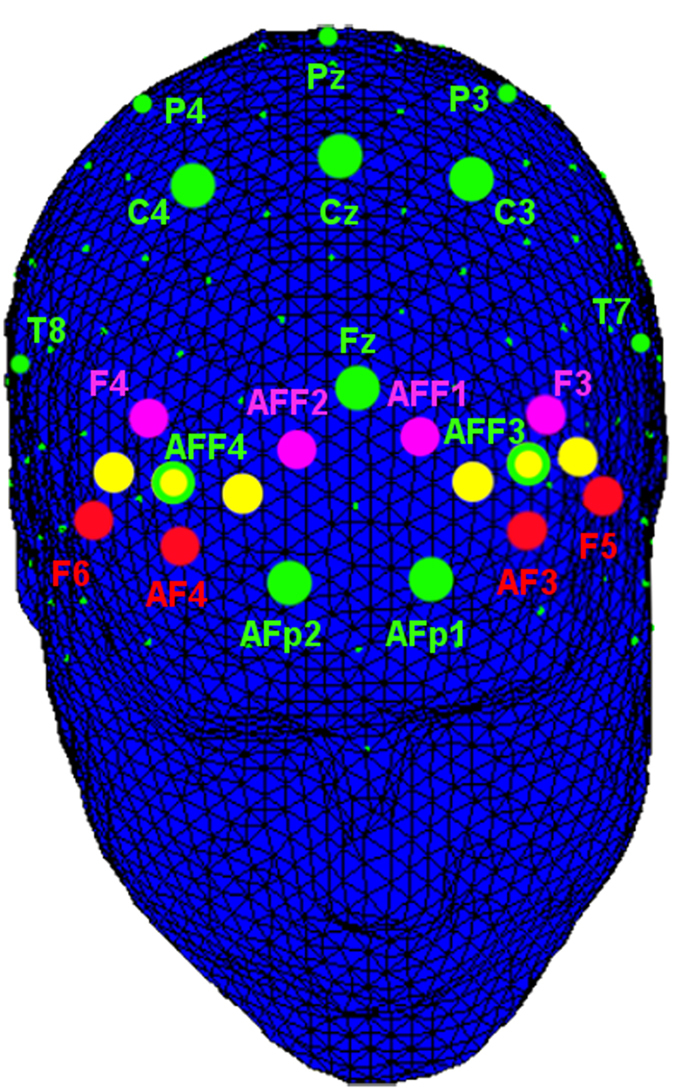
Locations of measurement channels. fNIRS: Emmiters were placed on positions AF3-AF4 and F5-F6 (red dots), while detectors were placed on AFF1-AFF2 and F3-F4 (pink dots). The 6 resulting channels are displayed with yellow dots. EEG: EEG activity was recorded from channels on the following positions: AFF3, AFF4, Fz, AFp1, AFp2, C3, C4, Cz, P3, P4, Pz, T7, T8, O1, O2 (green dots). fNIRS optodes and EEG channels were attached to the subject’s head using a NIRS-EEG compatible cup, with respect to the international 10/5 system.

A 32-channel portable EEG-System (V-AMP: Brain Products, München) was used for data acquisition. A NIRS-EEG compatible ElectroCap with Ag/AgCl electrodes was used to record EEG from active scalp sites referred to earlobe (10/5 system of electrode placement). EEG activity was recorded from channels on the following positions: AFF3, AFF4, Fz, AFp1, AFp2, C3, C4, Cz, P3, P4, Pz, T7, T8, O1, O2 (Fig. 6). The cap was fixed with a chin strap to prevent shifting during the task. The data were recorded during the stimulation using sampling rate of 500 Hz, with a frequency band of 0.01–50 Hz and with a notch filter of 50 Hz. The impedance of recording electrodes was monitored for each subject prior to data collection and it was always kept below 5 kΩ. Additionally, one EOG electrodes was sited on the outer canthus to detect eye movements. Ocular artefacts (eye movements and blinks) were corrected using an eye-movement correction algorithm that employs a regression analysis in combination with artifact averaging. After performing EOG correction and visual inspection, only artifact-free trials were considered (rejected epochs, 4%). The signal was visually scored, and portion of the data that contained artifacts were removed to increase specificity. Artifact-free epochs (850 ms) were considered. An averaged waveform (off-line) was obtained for each condition (not less than 22 epochs were averaged). The peak amplitude (higher peak amplitude from the baseline) was quantified relative to the 150 ms pre-stimulus. The onset was coincident with the appearance of the stimulus on the monitor, taking into account the most negative peak-value within the temporal window of 150–250 ms post-stimulus, since the morphological analysis of EEG profile revealed that the peak deflection was within this time range. Since the latency measure was previously tested without showing significant differences across condition, we did not include this variable within the final analysis. The mean latency of N200 was approximately 210 ms. Successively, localization (three sites: frontal, AFF3/AFF4 and AFp1/AFp2; temporo-central, C3/C4 and T7/T8 and parietal, P3/P4 and P7/P8) and lateralization (two sides: left channels and right channels) factors were considered to apply statistical analyses.

### fNIRS

fNIRS measurements were conducted with the NIRScout System (NIRx Medical Technologies, LLC. Los Angeles, California) using a 6-channel array of optodes (4 light sources/emitters and 4 detectors) covering the prefrontal area. Emmiters were placed on positions AF3-AF4 and F5-F6, while detectors were placed on AFF1-AFF2 and F3-F4. Emitter-detector distance was 30 mm for contiguous optodes and near-infrared light of two wavelengths (760 and 850 nm) was used. NIRS optodes were attached to the subject’s head using a NIRS-EEG compatible cup, with respect to the international 10/5 system^88^ (Fig. 6).

With NIRStar Acquisition Software, changes in the concentration of oxygenated (O2Hb) and deoxygenated haemoglobin (HHb) were recorded from a 120 s starting resting phase. Signals obtained from the 6 NIRS channels were measured with a sampling rate of 6.25 Hz, and analyzed and transformed according to their wavelength and location, resulting in values for the changes in the concentration of oxygenated and deoxygenated hemoglobin for each channel. Hemoglobin quantity is scaled in mmol∗mm, implying that all concentration changes depend on the path length of the NIR light in the brain.

The raw data of O2Hb, and HHb from individual channels were digitally band-pass filtered at 0.01–0.3 Hz. Then, the mean concentration of each channel within a subject was calculated by averaging data across the trials from the trial onset for 6 s. Based on the mean concentrations in the time series, we calculated the effect size in every condition for each channel within a subject. The effect sizes (Cohen’s d) were calculated as the difference of the means of the baseline and trial divided by the standard deviation (Sd) of the baseline: d = (m1−m2)/s. Accordingly, m1 and m2 are the mean concentration values during the baseline and trial, and s means the Sd of the baseline. Then, the effect sizes obtained from the 6 channels were averaged in order to increase the signal-to-noise ratio. Although the raw data of NIRS were originally relative values and could not be averaged directly across subjects or channels, the normalized data such as the effect size could be averaged regardless of the unit^89^,^90^,^91^. In fact, the effect size is not affected by differential pathlength factor (DPF)^90^.

## Additional Information

**How to cite this article**: Balconi, M. and Vanutelli, M. E. Hemodynamic (fNIRS) and EEG (N200) correlates of emotional inter-species interactions modulated by visual and auditory stimulation. *Sci. Rep.* **6**, 23083; doi: 10.1038/srep23083 (2016).
